# An Outbreak of Respiratory Viral Infections in a Professional Ice Hockey Team

**DOI:** 10.1111/irv.70041

**Published:** 2024-10-31

**Authors:** Wilma Grönroos, Petri Helenius, Maarit Valtonen, Matti Waris, Olli J. Heinonen, Olli Ruuskanen

**Affiliations:** ^1^ Paavo Nurmi Centre and Unit for Health and Physical Activity University of Turku Turku Finland; ^2^ Finnish Institute of High Performance KIHU Jyväskylä Finland; ^3^ Institute of Biomedicine, University of Turku and Department of Clinical Microbiology Turku University Hospital Turku Finland; ^4^ Department of Pediatrics and Adolescent Medicine Turku University Hospital Turku Finland

**Keywords:** athlete, exercise, ice hockey, influenza, respiratory virus, sport

## Abstract

**Background:**

Viral acute respiratory infections (ARIs) are an important cause of illness in athletes. However, their impact on ice hockey players is unclear.

**Method:**

We describe an outbreak of ARIs in a professional ice hockey team.

**Results:**

Contrary to expected influenza, the 40‐day outbreak was caused by 8 different respiratory viruses, that is, 2 different influenza A viruses, human coronavirus‐NL63 (HCoV‐NL63), respiratory syncytial viruses (RSV) A and B, 2 different rhinoviruses, enterovirus D68, and parainfluenza type 2 virus.

**Conclusion:**

Only influenza A and HCoV‐NL63 were possibly spread within the team thus suggesting an important contraction from the community. The burden of illness was substantial.

## 1 Introduction

Ice hockey is an indoor team sport characterized by short‐term maximal exercises and recurrent physical contact. Ice hockey season occurs at a time when the community's respiratory virus activity is high [1]. The game offers an ideal setting for the transmission of viral ARIs as players have maximal breathing, hugging, and shouting, and they sit closely side‐by‐side during breaks and in locker rooms [2, 3]. However, previous studies on the occurrence and transmission of viral ARIs in ice hockey are few and limited [4, 5, 6, 7].

This study was initiated to control a potential influenza outbreak within a single professional ice hockey team after several players fell ill during an influenza epidemic. We describe the occurrence, etiology, and clinical manifestations of viral ARIs in the players of the team. To describe the burden of illness, we report the games missed due to viral ARIs.

## 2 Material and Methods

The study population consists of a single professional ice hockey team playing in the Finnish National Hockey League. The team had 33 players and 11 staff members. One player was not included in the study because a nasal swab could not be taken. The prospective follow‐up study commenced on February 2, 2020, when two players fell ill. It was concluded on March 13 (40 days later) when the ice hockey season was prematurely suspended due to the onset of COVID‐19. The first COVID‐19 infection was detected in Helsinki, Finland, on February 26, and public preventive measures were initiated on March 12.

During the follow‐up period, all team members were reinforced to immediately report possible respiratory symptoms to the team physician (P.H.). At the onset of a symptom, a nasal mucus specimen was collected at a depth of 4–5 cm using flocked nasal swabs (503CS01, Copan Flock Technologies, Brescia, Italy). On February 15, a nasal swab was taken from all the team members.

A commercial rapid antigen detection test for influenza A and B viruses was used. When the first tests were negative, the following samples were studied in Turku University Hospital virus laboratory by a multiplex PCR test (AllPlex Respiratory Panels 1–4, Seegene, Seoul, South Korea): respiratory syncytial virus A and B, adenovirus, influenza A and B viruses, rhinovirus, enteroviruses, parainfluenza type 1–4 viruses, human coronaviruses 229E, OC43, and NL63, human bocavirus, and human metapneumovirus. In addition, rhinoviruses and enteroviruses were detected by triplex RT‐qPCR and typed by gene sequencing [8].

## 3 Results

**FIGURE 1:**
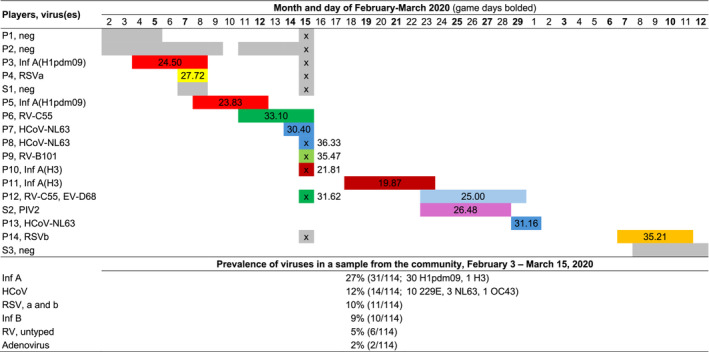
Timeline of respiratory viral infections in a professional ice hockey team. P, player; S, staff; Neg, virus negative; Inf A, influenza A virus; RSV A, respiratory syncytial virus A; RV, rhinovirus; HCoV NL63; human coronavirus NL63; EV, enterovirus; RSV B, respiratory syncytial virus B; PIV2, parainfluenza virus 2. Coloring, coded according to the virus finding, indicates symptomatic days over 2 or more days, and test result in an asymptomatic subject is marked with x. Numbers indicate PCR Ct values. Additional 11 players and 5 staff members were tested negative on February 15. Prevalence of respiratory viruses in the community according to the sentinel testing report from the Finnish Institute of Health and Welfare.

On February 2, 2020, 2 players (P1 and P2 in Figure 1) developed a fever and a cough. The players' nasal mucus samples tested negative for influenza in the antigen test. The symptoms lasted for 4 and 11 days. The players had to miss 2 games and 4 games, respectively. On February 4, P3 reported a sore throat and myalgia. Influenza A (H1pdm09) was diagnosed in the laboratory. The symptoms lasted for 3 days, and he missed 2 games. On February 7, P4 experienced congestion. Respiratory syncytial virus A (RSV A) was diagnosed by PCR. The symptoms lasted for 1 day and he missed 1 game. On February 8, P5 developed a fever, a sore throat, a cough, and a runny nose. Influenza A (H1pdm09) was detected. The symptoms lasted for 5 days and he missed 2 games. On February 11, P6 developed a sore throat and a cough. Rhinovirus (RV‐C55) was diagnosed. The symptoms lasted for 5 days and he missed 2 games. On February 14, P7 reported a nasal congestion. Human coronavirus NL63 (HCoV‐NL63) was detected. The nasal congestion persisted for 2 days; however, the player did not miss any games.

On February 15, nasal mucus samples were taken from all asymptomatic team members (27 players and 8 staff members). Four players (11%) were virus‐positive, 1 HCoV‐NL63 (P8), 1 RV‐B101 (P9), 1 influenza A subtype H3 (P10), and 1 RV‐C55 (P12). The players were considered asymptomatic. One player with a rhinovirus had experienced congestion 2 weeks earlier. The other player with an influenza A virus had experienced congestion in February and the player with HCoV‐NL63 had experienced a runny nose during the preceding week.

On February 18, P11 developed a fever, a cough, and myalgia. Influenza A (H3) was diagnosed. The symptoms lasted for 6 days and he missed 3 games. On February 23, P12 reported a cough and a runny nose. Enterovirus (D68) was detected from a nasal mucus sample in the laboratory. The symptoms lasted for 7 days and the player missed 3 games. On February 29, P13 experienced a fever and a runny nose. HCoV‐NL63 infection was diagnosed. The symptoms lasted for 1 day and the player missed 1 game. On March 8, P14 developed a runny nose. RSV B was detected in the nasal mucus. The symptoms lasted for 4 days but he did not miss any games.

During the study period, 3 staff (S1–S3) members experienced symptoms of ARI. One had a parainfluenza virus type 2 infection detected on 24 March and the other 2 were virus‐negative in the multiplex PCR test.

Altogether, 14 symptomatic ARIs (11 of 32 players) were recorded in the team; 49 nasal swabs were tested for viruses by multiplex PCRs, 14 from symptomatic and 35 from asymptomatic subjects. In the symptomatic subjects, 10 of the 14 PCR tests were positive (71%) identifying 8 different viruses. Six subjects had a high viral load, that is, a cycle threshold (Ct)‐value < 27 (Figure 1). Eleven players missed 20 games due to the viral ARIs during 40 days. In addition, 8 players missed 40 training days. Two players did not miss any training days.

## 4 Discussion

Our study has 3 main observations. First, viral diagnostics in ice hockey players with ARI proved beneficial. Second, the viral ARIs originated from the community, and only 2 viruses possibly spread within the team. Third, the disease burden of viral ARIs was substantial.

Virus diagnostics played a significant role during the outbreak. Febrile ARIs were originally thought to be influenza because the outbreak occurred during the influenza season (Figure 1). However, the 2 first ARI cases were influenza antigen test negative. Influenza rapid antigen tests may not be sensitive enough in adults [9]. Finally, of the 11 symptomatic ARIs, 3 cases were caused by influenza A virus, all identified by PCR. Influenza outbreaks in a sports team are important to diagnose because early administered oseltamivir treatment reduces the duration of symptoms and oseltamivir prophylaxis may prevent transmission [9, 10]. We recently reported influenza outbreaks detected by rapid antigen tests in 2 ice hockey teams. The outbreaks were effectively controlled by oseltamivir treatment and prophylaxis [7]. Recently evolved point‐of‐care antigen and PCR viral testing may have a place in the health care of athletes [11].

Contrary to the sole influenza A outbreak the ARIs proved to be caused by 8 different respiratory viruses (Figure 1). The probable etiology of infections could be detected in 82% of the symptomatic players. The transmission of ARIs within the team was limited. Only the possible spread of influenza A H3 virus and HCoV‐NL63 (to 1 player respectively) was observed suggesting that most viral ARIs were community‐acquired. This is in agreement with our observations during the 2018 Winter Olympic Games. During 21 days, 9 different respiratory virus infections were identified suggesting contraction from the outside community. In this study, only 1 infected player had children aged less than 5 years, who are a major risk factor for virus transmission [9, 12]. This player, however, was the only one identified with two ARIs. A plausible explanation for the limited intra‐team transmission of viruses could be reinforced prevention measures. The players also refrained from participating in practices and games during symptomatic periods. In addition, an HEPA air cleaner was installed in the locker room [13].

Contracting an ARI resulted in players of missing, an average, 1.8 out of 15 games. The ARI absences had substantial implications for the team's functioning, particularly during the initial phase of the study, when several players were ill. Furthermore, at the same time, some players were also suffering from an injury, and some were participating in the national team tournament. However, it is of note that athletes may train and compete during mild viral ARIs [14, 15]. To our knowledge, fever is the only exemption from playing in the National Ice Hockey League (NHL).

In ice hockey, previous studies on the occurrence of ARIs are limited. A prospective study conducted in Poland reported that young active male ice hockey players experienced an average of 1.8 ARIs during a 19‐week study period [5]. In a prospective cohort study in the professional league in Norway, the average number of illnesses per player was 1.0 during the 31‐week competitive season [6]. SARS‐CoV‐2 infection was detected during a 2‐week period in 24 players from 2 opposing hockey teams [4].

This study has limitations. The number of viral ARIs was small, and we had no genomic evidence of the spread of influenza A or HCoV‐NL63. In addition, we could not trace the source viral ARIs of the players.

In conclusion, viral ARIs may induce a marked burden of illness in professional ice hockey teams. Only a few viruses spread within the team, suggesting an important contraction from the community. Basic infection control measures are necessary for individual players as community members and within the teams during viral highs in the community.

## Author Contributions

**Wilma Grönroos:** conceptualization, writing – original draft, data curation, validation, formal analysis, visualization. **Petri Helenius:** conceptualization, investigation, writing – review and editing, data curation. **Maarit Valtonen:** conceptualization, writing – review and editing. **Matti Waris:** conceptualization, investigation, visualization, writing – review and editing. **Olli J. Heinonen:** conceptualization, writing – review and editing. **Olli Ruuskanen:** conceptualization, writing – review and editing, funding acquisition, project administration, methodology, supervision, data curation, validation, formal analysis.

## Ethics Statement

This study complied with the Declaration of Helsinki as revised in 2000, and all study‐related activities were conducted according to Good Clinical Practice. The study protocol was approved by the Ethics Committee of the Hospital District of Southwest Finland (ETMK Dnro: 5/1801/2019).

## Conflicts of Interest

The authors declare no conflicts of interest.

### Peer Review

The peer review history for this article is available at https://www.webofscience.com/api/gateway/wos/peer‐review/10.1111/irv.70041.

## Data Availability

The data that support the findings of this study are available from the corresponding author upon reasonable request.
